# The evidence for the physiological effects of lactate on the cerebral microcirculation: a systematic review

**DOI:** 10.1111/jnc.14633

**Published:** 2019-01-24

**Authors:** Tristan R. Hollyer, Luca Bordoni, Birgitte S. Kousholt, Judith van Luijk, Merel Ritskes‐Hoitinga, Leif Østergaard

**Affiliations:** ^1^ Centre for Functionally Integrative Neuroscience (CFIN) Aarhus University Aarhus C Denmark; ^2^ Institute for Clinical Medicine Aarhus N Denmark; ^3^ Department of Biomedicine South Aarhus University Aarhus C Denmark; ^4^ Department of Clinical Medicine AUGUST Centre Aarhus University Risskov Denmark; ^5^ SYstematic Review Centre for Laboratory Animal Experimentation (SYRCLE) Department for Health Evidence Radboud University Medical Centre Nijmegen The Netherlands; ^6^ Department of Neuroradiology Aarhus University Hospital Aarhus C Denmark

**Keywords:** brain, cerebral blood flow, lactate, microcirculation, systematic review

## Abstract

**Abstract:**

Lactate's role in the brain is understood as a contributor to brain energy metabolism, but it may also regulate the cerebral microcirculation. The purpose of this systematic review was to evaluate evidence of lactate as a physiological effector within the normal cerebral microcirculation in reports ranging from *in vitro* experiments to *in vivo* studies in animals and humans. Following pre‐registration of a review protocol, we systematically searched the PubMed, EMBASE, and Cochrane databases for literature covering themes of ‘lactate’, ‘the brain’, and ‘microcirculation’. Abstracts were screened, and data extracted independently by two individuals. We excluded studies evaluating lactate in disease models. Twenty‐eight papers were identified, 18 of which were *in vivo* animal experiments (65%), four on human studies (14%), and six on *in vitro* or *ex vivo* experiments (21%). Approximately half of the papers identified lactate as an augmenter of the hyperemic response to functional activation by a visual stimulus or as an instigator of hyperemia in a dose‐dependent manner, without external stimulation. The mechanisms are likely to be coupled to NAD ^+^/NADH redox state influencing the production of nitric oxide. Unfortunately, only 38% of these studies demonstrated any control for bias, which makes reliable generalizations of the conclusions insecure. This systematic review identifies that lactate may act as a dose‐dependent regulator of cerebral microcirculation by augmenting the hyperemic response to functional activation below 5 mmol/kg, and by initiating a hyperemic response above 5 mmol/kg.

**Open Science Badges:**

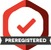

This article has received a badge for *Pre‐registration* because it made the data publicly available. The data can be accessed at www.radboudumc.nl/getmedia/53625326-d1df-432c-980f-27c7c80d1a90/THollyer_lactate_protocol.aspx. The complete Open Science Disclosure form for this article can be found at the end of the article. More information about the Open Practices badges can be found at https://cos.io/our-services/open-science-badges/.

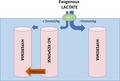

**Abbreviations used**
BOLD: blood‐oxygen‐level dependent
CAT: computer‐assisted tomography
CBF: cerebral blood flow
COX: cyclooxygenase
HCA1: hydroxycarboxylic acid receptor 1
HIF‐1α: hypoxia inducible factor‐1α
LDH: lactate dehydrogenase
MCT: monocarboxylate transporter
MeSH: medical subject headings
MRI: magnetic resonance imaging
NAD^+^: nicotinamide adenine dinucleotide
NADH: reduced nicotinamide adenine dinucleotide
NMRS: nuclear magnetic resonance spectroscopy
NOS: nitric oxide synthase
PET: positron emission tomography
PGE_2_: prostaglandin E_2_
RoB: risk of bias
VEGF: vascular endothelial growth factor

Lactate is an ubiquitous molecule in mammalian systems produced solely by lactate dehydrogenase (LDH), from pyruvate and reduced nicotinamide adenine dinucleotide (NADH) (Veech 1991). Its role in the brain is typically associated with neural energetics and the controversial astrocyte‐neuron lactate shuttle hypothesis (Dienel 2011, 2017; Mächler *et al*. 2016). Lactate is the endogenous agonist of the hydroxycarboxylic acid‐1 (HCA1) G‐protein coupled receptor, present on endothelial cell membranes, pericytes, astrocytes, and synaptic spines (Blad *et al*. 2011; Lauritzen *et al*. 2014; Morland *et al*. 2017). Evidence from our laboratory indicates that lactate is produced as a consequence to differential transport of glucose and oxygen across the blood–brain barrier (Angleys *et al*. 2016).

In brain tissue, proton‐coupled lactate transport via monocarboxylate transporters (Bergersen 2015) and lactate exchange via ion channels (Sotelo‐Hitschfeld *et al*. 2015; Karagiannis *et al*. 2016; Hadjihambi *et al*. 2017) suggest a broader role of lactate as a signaling molecule. Accordingly, lactate modulates neuronal excitability (Sotelo‐Hitschfeld *et al*. 2015) and is also thought to act as a volume transmitter, coordinating energy metabolism and blood flow in the brain and other organs, possibly via mechanisms that involve NADH and hence cellular redox state (Bergersen and Gjedde 2012; Mosienko *et al*. 2015; Proia *et al*. 2016). Given the complexity of the mechanisms that control cerebral blood flow (CBF) at a microvascular level (Attwell *et al*. 2010; Hall *et al*. 2014) it is crucial to better understand the vascular effects of lactate.

Progress in biomedical research is impeded if studies are underpowered or experimental procedures incompletely reported, as this increases the risk of positive reporting bias (Macleod *et al*. 2015). The resulting poor reproducibility, in turn, is a source of wasted resources (Freedman *et al*. 2015). With greater attention focusing toward the translational efficacy and reporting quality of pre‐clinical research, it is pertinent to consider evaluating existing literature prior to conducting *in vivo* experiments. This approach also sustains the 3R principles of Replacement, Reduction, and Refinement (Russell and Burch 1959). Moreover, it has been emphasized that systematic meta‐research should be conducted to identify factors contributing to high translational ability of scientific findings, as well as to help demarcate the ideal ratio between basic and applied research to achieve these aims (Chalmers *et al*. 2014). Systematic reviews provide a means of identifying trends in the existing research pool, of improving the quality and translational efficacy of studies, and of identifying unaddressed aspects in experimental design, which otherwise create a risk of bias (Hooijmans and Ritskes‐Hoitinga 2013; Ritskes‐Hoitinga and Wever 2018). Systematic reviews therefore help drawing more reliable conclusions from the existing literature while identifying ways of improving the design of future works.

This systematic review investigates the current evidence for how the cerebral microvasculature responds to lactate in studies ranging from the cellular level to human experiments. Using this broad scoped approach, we aimed to expand the systematic review paradigm with a focus on intervention studies, to demonstrate that novel extractions of existing data help guide the formulation of new hypotheses.

## Materials and methods

### Review protocol & amendments

The protocol for this systematic review was pre‐defined using the SYRCLE guidelines (de Vries *et al*. 2015) and published on www.radboudumc.nl/en/research/radboud-technology-centers/animal-research-facility/systematic-review-center-for-laboratory-animal-experimentation/protocols on 5th December 2017 prior to completion of primary screening. Post‐publication modifications were made to the protocol as follows: (i) At the primary‐screening phase, discrepancies on decision to include were resolved by Tristan R Hollyer (TRH) and Birgitte S Kousholt (BSK). (ii) At the end of primary screening, Luca Bordoni (LB) was recruited to conduct the full text screening and data extraction as BSK and Judith van Luijk (JvL) were unable to contribute to these processes further. (iii) Discrepancies on decision at the full‐text screening phase were resolved by TRH and LB. (iv) Leif Østergaard (LØ) was included as a contributing author. (v) A modified number of risk of bias measures were decided upon and then evaluated by TRH as described below.

During the data extraction process, we identified several studies which evaluated the effect of lactate on cerebral blood flow by different experimental measurements both in animal and human studies. We decided to extract these datasets in an aim to identify potential trends in findings. To further determine the methodological quality of these extracted studies, TRH assessed them for risk of bias (RoB) by determining if each study reported the use of bias limiting measures such as population randomization, blinding, or sample size calculation.

### Study search, selection, screening, and extraction

The PubMed, EMBASE, and Cochrane databases were systematically searched electronically on 14th October 2017. The search strategy was comprised of three categories: ‘lactate (and related enzymes, transporters, and receptors)’, ‘microvasculature’, and ‘brain’. Within each category, medical subject headings terms were determined and relevant synonyms were sought within titles, abstracts, and keywords. The full search strategy is detailed in Table  S1. No restrictions were applied to language or publication date. The search results were pooled, with duplicates removed, and indexed in EndNoteX8 Software (Clarivate Analytics, Philadelphia, PA, USA). Original articles and clinical trials were included, and review articles excluded. The library was then uploaded to the online systematic review management platform Covidence (http://covidence.org).

As stated in the study protocol, studies were included if they examined lactate's role in cerebral circulation only in physiological conditions. Where disease/pathological models were used, we extracted data from appropriate controls whenever available. Selected populations ranged from endothelial cell lines, *ex vivo* tissue, *in vivo* animal, and human studies. Interventions were defined as any modification of lactate or its pharmacological effectors, for example, receptors, transporters, generating enzymes (LDH) and any non‐harmful genetic modification, for example, receptor knock‐outs, or relevant control or baseline data. Defined outcomes were any stated measures related to the effects of the experimental treatments on population biochemistry or physiology; cerebral vascular cell/tissue behavior (such as diameter or flow behavior) evaluated directly or indirectly.

Titles and abstracts were screened for inclusion independently by TRH and BSK/JvL, the latter at a proportion of 80/20%. Disputes were resolved by TRH and BSK. The reference sections of all texts selected for full‐text screening were checked for additional references of interest. Studies included for full‐text screened were evaluated, and data extracted independently by TRH and LB.

Extracted studies are summarized in characteristics tables (Tables S2–S4). In each table, the author, publication data, species, strain, age of tissue source/animal/subject; number of experimental units, intervention method, assessment method, and primary findings are outlined. For ease of interpretation, lactate concentrations were converted to mmol/kg whenever possible, and blood plasma lactate values converted to mmol/L.

## Results

**Figure 1:**
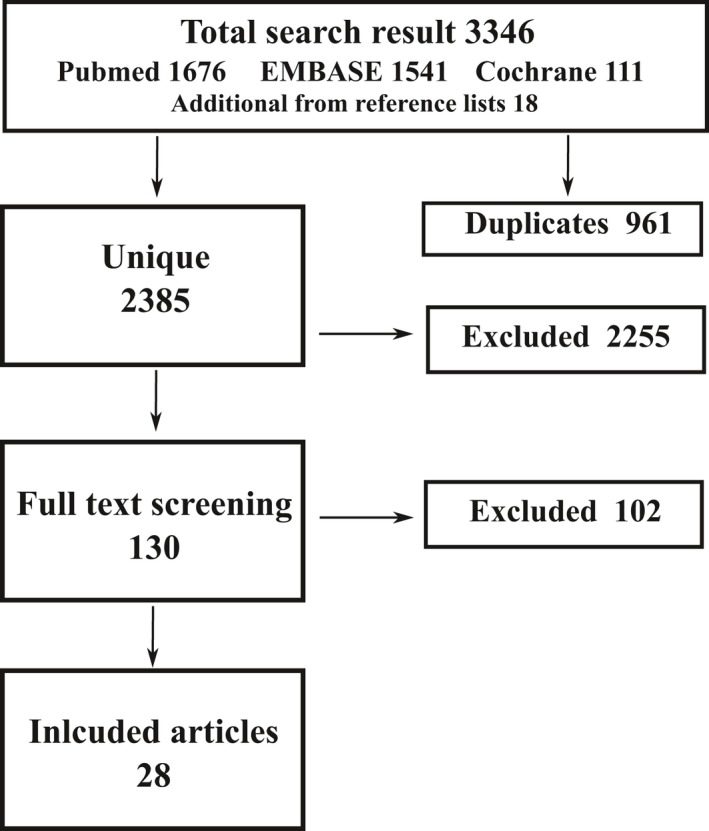
Flow diagram of the study selection and screening process.

The selection process is illustrated in Fig. 1 and the full search strategy is shown in Table  S1. The search in the PubMed, Embase, and Cochrane databases identified a total of 2385 unique references of which 130 were assessed for eligibility for data extraction. From within their references sections, an additional 18 sources were identified. Of the studies assessed, 28 (nine of which were found from references) were included in the final review.

### Study characteristics

The characteristics of all selected publications are detailed in Tables S2–S4 indicating the model used; the intervention used; the methods to assess lactate concentrations or model responses, for example, CBF; and a summary of the main findings. Of the 28 studies included, six were *in vitro* or *ex vivo* experiments (21%), 18 were *in vivo* animal experiments (65%), and four human studies (14%).

### *In vitro*/*ex vivo* studies

Of the *in vitro* and *ex vivo* studies (Table  S2), the two earliest works demonstrated an age‐dependent relationship between the presence of LDH and lactate uptake by cortical vessels (Spatz *et al*. 1978; Rieke and Cannon 1985). Detailed anatomical investigations by Lauritzen *et al*. (2014) identified the presence of the HCA1 receptor on the luminal and abluminal membranes of the mouse endothelial cell at a density twice as that on astrocytic end‐feet. Cellular responses to exogenous lactate were studied separately twice. Sub‐physiological levels of lactate had no effect on cell survival (Pirchl *et al*. 2006), but 20 mmol/L lactate applied to human brain endothelial cells induced a marked response to cellular lactate uptake and cellular proliferation (Miranda‐Gonçalves *et al*. 2017). Gordon *et al*. (2008) was the only *ex vivo* study that evaluated the pharmacological mechanisms behind lactate signaling in rat arterioles. It was found that lactate induces arteriolar dilation by reducing the reuptake of PGE_2_ at astrocyte end‐feet, allowing for continued PGE_2_ binding to prostaglandin receptors on vascular smooth muscle cells.

### *In vivo* studies

**Figure 2:**
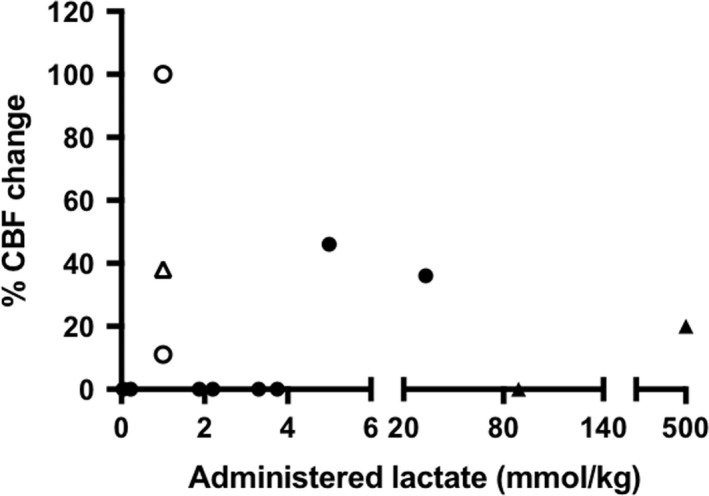
The percentage cerebral blood flow (CBF) response to lactate administration in both animals and humans. Administered concentrations below 5 mmol/kg will only augment the CBF response to stimulus (clear symbols). Higher doses of lactate elicited a CBF response in the absence of stimulation (dark symbols). Animal studies (circle), human studies (triangle). Details of individual studies in Table 1.

The majority of *in vivo* animal studies (Table  S3) were conducted in mongrel dogs (Harper and Bell 1963; Iwabuchi *et al*. 1973; Hermansen *et al*. 1984; Young *et al*. 1991), or rats (Hallström *et al*. 1990; Ido *et al*. 2001, 2004; Provent *et al*. 2007). Almost half (10) of the *in vivo* studies evaluated the effect of systemic administration of lactate (either as an acid or its sodium salt) on CBF in animals. To elucidate any trends in these findings, results from these experiments were evaluated in combination with similar human studies (see below, Fig. 2 and Table  S3).

Lactate only induced a hyperemic response (identified via radiolabeling techniques) if administered in doses of above 5 mmol/kg (Bucciarelli and Eitzman 1979; Young *et al*. 1991), with resulting blood plasma concentrations of 30 mmol/L reported in Young *et al*. (1991). Studies infusing lactate at lower concentrations found no direct hyperemic response. However, in both rats and non‐human primates, an increase in blood plasma lactate concentrations of 2.6 ± 0.0 and 3.5 ± 0.4 (Ido *et al*. 2004), and 2.5 ± 0.9 mmol/L (von Pföstl *et al*. 2012), led to an augmentation of the CBF response to visual stimuli.

The long‐term effects of both exhaustive exercise and repeated administration of lactate were studied in two separate experimental groups of mice (Lezi *et al*. 2013; Morland *et al*. 2017). Both studies found that increased plasma lactate levels brought about by both exercise and systemic administration induced a comparable increase in brain vascular endothelial growth factor‐A (VEGF‐A) and their related signaling pathways. Morland *et al*. (2017) subsequently found that VEGF‐A had a pro‐angiogenic effect in both the hippocampus and sensorimotor cortex.

### Human studies

Selected human studies are characterized in Table  S4. Two of these studies (Stewart *et al*. 1988; Reiman *et al*. 1989) evaluated how lactate infusion (a reported anxiolytic) influenced CBF in patients with anxiety disorders and control subjects. The latter showed a mean increase in CBF of 20% when administered 500 mmol/kg lactate (Stewart *et al*. 1988) but no CBF change when given a lower concentration (up to 133 mmol/kg) (Reiman *et al*. 1989). Mintun *et al*. (2004), infused 1 mmol/kg lactate, increasing blood plasma concentrations to 10.7 ± 2.8 mmol/L, and observed unaltered resting CBF, but augmented CBF response to stimulation, by up to 53%.

### Effects of lactate on CBF across species

We identified 10 *in vivo* animal and 3 human publications which evaluated the effect of direct systemic administration of lactate on CBF. To elucidate any trends in findings, results from these experiments were extracted and evaluated as shown in Fig. 2 (details are presented in Table 1). At concentrations of over 5 mmol/kg, lactate‐induced cerebral hyperemia. At concentrations lower than 5 mmol/kg, lactate augmented CBF response to stimuli.

**Table 1:** Selected *in vivo* and human studies which reported cerebral blood flow measurements when administering lactate systemically

Authors	Species	Salt/Acid	Dose (mmol/kg)	Delivery	Anesthesia (neuromuscular agents)	CBF assessment method	Reported response	Notes
Bucciarelli and Eitzman (1979)	Goat	Acid	5–10	IV	Chloralose	Radiolabeled microspheres	+46%	Plasma lactate not reported
Dostalova *et al*. (2018)	Rabbit	Salt	1.87	IV	Isoflurane and fentanyl (pipecuronium)	Side stream dark‐field	No change	
Harper and Bell (1963)	Dog	Acid	0.22 mmol/L	IA	Thiopentone (suxamethonium)	^85^Kr washout	No change	
Hermansen *et al*. (1984)	Dog	Acid	2.20	IV	Pentobarbitol (pancuronium)	Radiolabeled microspheres	No change	
Ido *et al*. (2001)	Rat	Salt	1.00	IV	Urethane	^125^I‐desmethylimipramine	+100%	Augmented stimulus response
Ido *et al*. (2004)	Rat	n/a	1.00	IV	Urethane	^125^I‐desmethylimipramine	+11%	Augmented stimulus response
Ong *et al*. (1986)	Sheep	Acid	3.30	IV	d‐Tubocurarine	^113^Xe washout	No change	
Powell *et al*. (1985)	Dog	Acid	3.75	IV	Halothane (pancuronium)	[^14^C]iodoantipyrine	No change	Once corrected for pCO_2_
von Pföstl *et al*. (2012)	Monkey	Salt	0.04	IV	Remifentanyl (mivacurium chloride)	MRI	No change	Detection threshold/augment BOLD signal
Young *et al*. (1991)	Dog	Acid	33.3 mol/L	IV	Halothane (pancuronium)	[^14^C]iodoantipyrine	+36%	Plasma lactate 30 mmol/L
Stewart *et al*. (1988)	Human	Salt	500	IV	n/a	Inhaled ^133^Xe CAT	+20%	Plasma lactate not reported
Reiman *et al*. (1989)	Human	Salt	89	IV	n/a	[^15^O] water PET	No change	
Mintun *et al*. (2004)	Human	n/a	1.00	IV	n/a	[^15^O] water PET	+38%	Augmented stimulus response

BOLD, blood‐oxygen‐level dependent; CBF, cerebral blood flow; CAT, computer‐assisted tomography; IV, intravenous; IA, intra‐arterial; MRI, magnetic resonance imaging; PET, positron emission tomography.

The concentration of lactate (either as a sodium salt or acid), route of administration, anesthesia used, method of CBF evaluation, and reported effects are evaluated.

### Risk of bias assessment

We then conducted a modified assessment of reporting bias to evaluate the validity of these findings. Only one paper reported the use of blinding, randomization and sample size calculation methods to control for bias (Dostalova *et al*. 2018). Two reported the use of randomizing of subjects to treatment (Bucciarelli and Eitzman 1979; Ong *et al*. 1986) and two of blinding subjects to treatment (Reiman *et al*. 1989; Mintun *et al*. 2004). The remaining papers did not state as to whether they took bias controlling measures.

## Discussion

Despite continued uncertainty into the precise mechanisms of flow metabolism coupling in the brain, the role of lactate, as an effector of microvessel behavior, has not been assessed. By providing a systematic summary of publications on how the microvascular system responds to lactate across various experimental platforms, we have provided insights into unexplored avenues of research and identified considerations that the reader should make when planning their own experiments.

### Evidence on biochemical regulation of cerebral microvessels by lactate

Experiments conducted *in vitro,* or *ex vivo* comprised only 21% of all selected studies. The earliest studies showed age and vessel size‐dependent responses to lactate uptake and production, respectively (Spatz *et al*. 1978; Rieke and Cannon 1985). The later anatomical study by Lauritzen *et al*. (2014) identified the presence of the HCA1 receptor in differing amounts within the neurovascular unit. In particular, they reported that twice as many receptors were found on endothelial cell membranes compared to astrocytic end‐feet, suggesting a greater sensitivity of cerebral microvessels to lactate than astrocytes. Furthermore, Miranda‐Gonçalves *et al*. (2017) showed that glucose uptake was down‐regulated in favor of lactate uptake in response to high extracellular concentrations of lactate in immortalized cells derived from of human brain endothelium. In addition, large increases in mitochondrial activity, cell migration, and formation of capillary‐like structures and associated pro‐angiogenic factors such as hypoxia inducible factor‐1α (HIF‐1α), a transcriptional regulator of VEGF‐A occurred (Semenza 2010). At the arteriolar level, Gordon *et al*. (2008) showed that 1 mM lactate‐induced arteriolar dilation via a cyclo‐oxygenase (COX) dependent manner, further complementing earlier evidence (Kaidi *et al*. 2006; Benderro and LaManna 2014) that HIF‐1α is a key regulator of COX‐2. Alongside the findings of Morland *et al*. (2017), that repeated exposure to lactate, artificially or through exercise, promotes VEGF expression, it appears that lactate exerts both short‐ and long‐term angiogenetic effects on the cerebral microvascular in via a common mechanism. Such responses are perhaps unsurprising, considering the classical view of lactate being produced during anaerobic glycolysis as a consequence of exercise or hypoxia. Therefore, it demonstrates that the pathways, which are up‐regulated in response to hypoxia and exercise, also have a role in cerebral vascular homeostasis and hemodynamics, ensuring that sufficient glucose and oxygen are delivered to the cells of the brain.

Intravenous administration of up to 2.0 mmol/L lactate has no effects on resting CBF, regardless of choice of anesthesia (Fig. 2 and Table  S3), but enhances the CBF response to stimulation in both animals and humans (Ido *et al*. 2001, 2004; Mintun *et al*. 2004). Indeed, CBF responses to functional activation seemingly correlate with arterial lactate/pyruvate ratios (Mintun *et al*. 2004), which is coupled to the NADH/NAD^+^ ratio.

**Figure 3:**
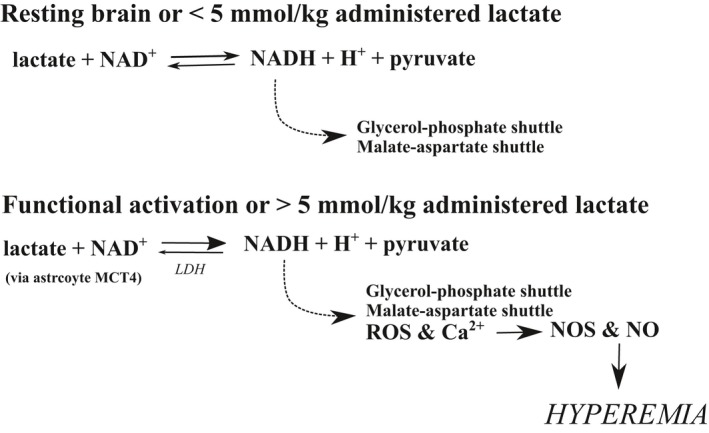
Reaction scheme illustrating the thresholds for which excess production of NADH (from lactate) alters redox state, inducing hyperemia during functional activation or administration of over 5 mmol/kg lactate.

Ido *et al*. (2001, 2004) hypothesized that the NADH/NAD^+^ ratio acts as sensor acting as a regulator of CBF when increased via activation of constitutive nitric oxide synthase. In their studies, the absence of any CBF response in the unstimulated regions of the brain is suggested to be due to exogenous lactate being oxidized to pyruvate (via LDH). This leads to a concurrent increase in NADH/NAD^+^ ratio which is balanced by transfer of NADH to the glycerol‐phosphate and malate‐aspartate shuttles (see Fig. 3) (Mintun *et al*. 2004). It is only when these pathways become saturated, due to the additive effect of > 5 mmol/kg lactate or functional activation (resulting in an accumulation of NADH), that a subsequent increase in reactive oxygen species results in elevated Ca^2+^ before recruitment the of nitric oxide synthase (NOS) pathways (Wolin 1996). Bucciarelli and Eitzman (1979), Stewart *et al*. (1988), and Young *et al*. (1991) (Table  S3), reported large increases in resting CBF following administration concentrations of lactate higher than 5 mmol/kg. It is therefore likely that the higher blood plasma concentrations of lactate in these experiments (as illustrated in Fig. 3), lead to much greater rises in the lactate/pyruvate ratio and NADH : NAD^+^ ratios, causing a greater accumulation of NADH and reactive oxygen species, leading to a much larger increase in CBF. However, Reiman *et al*. contradicts this hypothesis showing no CBF response. Furthermore, what was not explored was how cellular redox potential is driven also by glyceraldehyde‐3‐phosphate dehydrogenase and, also the phosphorylation state of the cytosolic adenine nucleotide system (Veech *et al*. 1970).

Monocarboxylate transporter 4 is the predominant monocarboxylate transporter on astrocytes (Bergersen 2015) with a *K*_m_ of 28 mmol/L (Manning‐Fox *et al*. 2000). This is indicative of astrocytes having a far greater capacity for lactate uptake than neurons (Dienel 2012). Changes in astrocytic NADH : NAD^+^ ratios has been linked to nitric oxide production by astrocytes (Buskila *et al*. 2005). However, transcriptome data (GOAD database, http://bioinf.nl:8080/GOAD2/databaseSelectServlet) suggest that astrocytes do not express NOS isoforms. Furthermore, calcium influx (also induced by oxidative stress) leads to activation of nNOS –derived NO in neurons and release of vasoconstrictors from astrocytes. Meanwhile, nNOS derived NO inhibits astrocytic COX‐2 and thus the production of astrocyte‐derived vasodilators (Attwell *et al*. 2010). Nitric oxide is also known to have positive reciprocal regulatory relationship with HIF‐1α (Poyton and Hendrickson 2015), whose relationship with lactate is described above.

Tissue pH is a powerful regulator of arterial tone (Yoon *et al*. 2012), and the augmented CBF observed in response to elevated lactate levels might therefore be related to parallel acidification via co‐transport of protons via monocarboxylate transporters, Experimental data suggest, however, that lactate‐induced CBF changes are caused by the higher lactate concentrations rather than the parallel changes in pH (Laptook *et al*. 1988).

One of the aim of this systematic review was to collectively analyze evidence showing that lactate can serve as a regulator of cerebral microvasculature. Although we lack direct experimental proofs to confirm this specific hypothesis, the current evidence points toward a coordinated system of local control of the cerebral microvasculature in which lactate is a key regulator with concentration specific thresholds for the magnitude of the response. This complements our own modeling of microvessel flow patterns which has shown that during functional hyperemia, glucose uptake is facilitated more so than oxygen favoring non‐oxidative glucose consumption suggesting that lactate may feedback into these control systems (Angleys *et al*. 2016).

### Considerations on RoB

Only five of the 13 papers (38%), which evaluated CBF responses to lactate, reported using methods to control for bias. As interest in research reproducibility increases, it is important that research which is exploratory in nature (regardless of the model) takes a robust approach to study design and control for bias (Kimmelman *et al*. 2014; Dirnagl 2016). We believe that our modified RoB assessment underscores the need for implementation of the systematic review methodology in basic science.

### Assumptions

During this review, we have made some assumptions about some of the data extracted. Several of the early studies administer lactic acid as a model of perinatal hypoxia under the hypothesis that lactic acidosis may have deleterious consequences to CBF or cerebral autoregulation (Harper and Bell 1963; Bucciarelli and Eitzman 1979; Hermansen *et al*. 1984; Powell *et al*. 1985; Ong *et al*. 1986). In this review we considered the induction of hyperlactemia (using lactic acid) as a non‐pathological or disease related intervention as long normoxia was maintained.

Contradictory literature reports may partly result from differences in the physiological mechanisms resulting in changes in lactate concentration due to the fact that multiple physiological situations may lead to alterations in lactate levels.

### Additional relevant literature

We wish to direct to the reader to the fact that, due to the methodology of this review, we noted that several papers provide insights into the effects of lactate on microvessels that are located outside the brain parenchyma. An elegant set of studies by Yamanishi *et al*. (2005) evaluated lactate's role in the function of retinal microvessels and pericytes and demonstrated that the contractile responses of pericytes to lactate were dependent on oxygenation of the preparation. Hein *et al*. (2006) also demonstrated this in porcine retinal arterioles dilated in response to lactate via nitric oxide synthase (NOS) pathways. However, it should be noted that the retina is a highly glycolytic environment (Winkler 1981) and as such, these studies may illustrate physiological responses unique to the retinal microvasculature. Cochlear capillaries have also been shown to dilate in response to lactate via NOS (Dai *et al*. 2011). It therefore may be likely that lactate acts (either directly or via NADH) on microvessels via a common nitric oxide‐dependent mechanisms across multiple systems.

Works by Rasmussen *et al*. (2006, 2009) were excluded on the basis that blood flow velocities were recorded from the middle cerebral artery, which is not a microvessel. However, they do report that lactate/pyruvate ratios (a representation of redox state) may be a regulating factor in CBF during activation and during the onset of exercise which is further supported by Vlassenko *et al*. (2006). The mechanisms highlighted in this review would benefit from replication and further investigation, in particular mechanisms which control the threshold at which lactate switches from an augmentor of hyperemia with a separate stimulus (e.g. visual) to an initiator (without another stimulus).

This review has used systematic literature search techniques to comprehensively assess existing evidence on the role of lactate in the cerebral microcirculation. Using systematic review methodology to probe questions of a fundamental physiological nature in studies ranging from *in vitro* experiments to human studies, allows us to fully appreciate the field in the entire research chain. This approach has identified that exogenous administered lactate may act as a regulator of cerebral blood flow in a dose‐dependent manner whereby at a threshold of 5 mmol/kg there is a switch from augmentation of the hyperemic response, to one of an initiator. We hope that this review provides a guide to the novel physiological properties of lactate in the brain, stimulates new interpretations of existing data, and highlights routes of exploration for further research.

## Supporting information


**Table S1.** Search strategies of the PubMed, Embase, and Cochrane databases.
**Table S2.** Characteristics of *in vitro* and *ex vivo* studies.
**Table S3.** Characteristics of selected *in vivo* animal studies.
**Table S4.** Characteristics of selected human studies.
