# Supplementary material for: The evidence for the physiological effects of lactate on the cerebral microcirculation: a systematic review
Source: J Neurochem. 2019 Jan 24;148(6):712–30. doi: 10.1111/jnc.14633 (PMC6590437; doi:10.1111/jnc.14633)
Supplement: Supplementary file 1 — Table S1. Search strategies of the PubMed, Embase, and Cochrane databases. Table S2. Characteristics of in vitro and ex vivo studies. Table S3. Characteristics of selected in vivo animal studies. Table S4. Characteristics of selected human studies. [file JNC-148-712-s001.pdf]

The evidence for the physiological effects of lactate on the cerebral microcirculation: a systematic review

Supplementary files

Tristan R. Hollyer[1,2], Luca Bordoni[3], Birgitte S. Kousholt[2,4], Judith van Luijk[5], Merel Ritskes-Hoitinga[5], Leif Østergaard[1,2,6]

Affiliations

[1] Centre for Functionally Integrative Neuroscience (CFIN), Aarhus University, Nørrebrogade 44, 8000 Aarhus C, Denmark

[2] Institute for Clinical Medicine, Palle Juul-Jensens Boulevard 99, 8200 Aarhus N, Denmark

[3] Department of Biomedicine South, Aarhus University, Wilhelm Meyers Allé 3, 8000 Aarhus C, Denmark

[4] Department of Clinical Medicine, AUGUST Centre, Aarhus University, 8240 Risskov, Denmark

[5] SYstematic Review Centre for Laboratory Animal Experimentation (SYRCLE), Department for Health Evidence, Radboud University Medical Centre, Geert Grooteplein-Noord 29, Route 231, 6525 GA Nijmegen, The Netherlands

[6] Department of Neuroradiology, Aarhus University Hospital, Nørrebrogade 44, 8000 Aarhus C, Denmark

Running Title: Role of lactate on cerebral microcirculation

Corresponding Author

Tristan R. Hollyer, Centre for Functionally Integrative Neuroscience (CFIN), Aarhus University, Nørrebrogade 44, 8000 Aarhus C, Denmark. Tel: +45 78 46 43 98, Fax: +45 78 46 44 00, Email: thollyer.research@gmail.com

Keywords: Systematic review, lactate, microcirculation, brain, cerebral blood flow

**Search Strategies**	
**PubMed**	“lactic acid"[MeSH Terms] OR lactic acid[tiab] OR lactate[tiab] OR (lactic[tiab] AND acid receptors[tiab]) OR (lactic[tiab] AND acid receptor[tiab]) OR GPR81[tiab] OR HCAR1[tiab] OR (Hydroxycarboxylic[tiab] AND acid receptor 1[tiab]) OR (“Monocarboxylic Acid Transporters"[MeSH Terms]) OR (monocarboxylic[tiab] AND transport*[tiab]) OR MCT[tiab] OR MCTs[tiab] OR MCT1[tiab] OR MCT2[tiab] OR MCT3[tiab] OR MCT4[tiab] OR ("lactate dehydrogenases"[MeSH Terms] OR "l-lactate dehydrogenase"[MeSH Terms]) OR lactate dehydrogenase[tiab] OR LDH[tiab] OR (hydroxypropanoic [tiab] AND acid[tiab]) AND "neurovascular coupling"[MeSH Terms] OR “neurovascular coupling”[tiab] OR neurovascular[tiab] OR neurovasculature[tiab] OR brain [MeSH] OR brain[tiab] OR brains[tiab] OR cerebral[tiab] OR "neurons"[MeSH Terms] OR neuron[tiab] OR neurons[tiab] OR neural[tiab] OR (nerve[tiab] AND (cell[tiab] OR cells[tiab])) OR "neuroglia"[MeSH Terms] OR ((glial [tiab] OR neuroglial [tiab]) AND (cell[tiab] OR cells [tiab])) OR "astrocytes"[MeSH Terms] OR astrocyte[tiab] OR astrocytes[tiab] OR astroglia [tiab] OR astroglias [tiab] OR astroglial [tiab] OR microglia[tiab] OR microglial [tiab] OR "pericytes"[MeSH Terms] OR pericyte[tiab] OR pericytes[tiab] OR (Rouget[tiab] AND (cell [tiab] OR cells [tiab])) AND "microvessels"[MeSH Terms] OR microvessel[tiab] OR microvessels[tiab] OR microvasculature[tiab]OR microvascular [tiab]OR ((Anastomoses[tiab] OR anastomosis [tiab]OR Anastomose [tiab]) AND Arteriovenous[tiab])) OR capillaries[tiab] OR capillary[tiab] OR Sinusoidal [tiab] OR sinusoids [tiab] OR sinusoid [tiab] OR arteriole[tiab] OR arterioles[tiab] OR venule[tiab] OR venules[tiab] OR "endothelium, vascular"[MeSH] OR endothelium[tiab] OR endotheliums [tiab] OR endothelial[tiab] OR “hemodynamics”[MeSH] OR ((hemodynamic[tiab] OR haemodynamic[tiab]) AND (response [tiab] OR responses [tiab]))
**Embase**	‘lactic acid’/exp OR ‘lactic acid’:ta,ab OR lactate:ta,ab OR (hydroxypropanoic:ta,ab AND acid:ta,ab) OR (lactic:ta,ab AND ‘acid receptors’:ta,ab) OR (lactic:ta,ab AND ‘acid receptor’:ta,ab) OR GPR81:ta,ab OR HCAR1:ta,ab OR (Hydroxycarboxylic:ta,ab AND ‘acid receptor 1’:ta,ab) OR (‘monocarboxylate transporter’/exp) OR (monocarboxylic:ta,ab AND ‘transport*’:ta,ab) OR MCT:ta,ab OR MCTs:ta,ab OR MCT1:ta,ab OR MCT2:ta,ab OR MCT3:ta,ab OR MCT4:ta,ab OR ‘lactate dehydrogenases’/exp OR ‘lactate dehydrogenase’:ta,ab OR ‘L-lactate dehydrogenase’:ta,ab OR LDH:ta,ab AND ‘neurovascular coupling’/exp OR ‘neurovascular coupling’:ta,ab OR neurovascular:ta,ab OR neurovasculature:ta,ab OR brain/exp OR brain:ta,ab OR brains:ta,ab OR cerebral:ta,ab OR ‘nerve cell’/exp OR neuron:ta,ab OR neurons:ta,ab OR neural:ta,ab OR (nerve:ta,ab AND (cell:ta,ab OR cells:ta,ab)) OR ‘glia’/exp OR ((glial:ta,ab OR neuroglia:ta,ab OR neuroglial:ta,ab) AND (cell:ta,ab OR cells:ta,ab)) OR ‘astrocytes’/exp OR astrocyte:ta,ab OR astrocytes:ta,ab OR astroglia:ta,ab OR astroglias:ta,ab OR astroglial:ta,ab OR microglia:ta,ab OR microglial:ta,ab OR ‘pericytes’/exp OR pericyte:ta,ab OR pericytes:ta,ab OR (Rouget:ta,ab AND (cell:ta,ab OR cells:ta,ab)) AND ‘microvasculature’/exp OR microvessel:ta,ab OR microvessels:ta,ab OR microvasculature:ta,ab OR microvascular:ta,ab OR ((Anastomoses:ta,ab OR anastomosis:ta,ab OR Anastomose:ta,ab) AND Arteriovenous:ta,ab) OR capillaries:ta,ab OR capillary:ta,ab OR Sinusoidal:ta,ab OR sinusoids:ta,ab OR

	sinusoid:ta,ab OR arteriole:ta,ab OR arterioles:ta,ab OR venule:ta,ab OR venules:ta,ab OR ‘vascular endothelium’/exp OR endothelium:ta,ab OR endotheliums:ta,ab OR endothelial:ta,ab OR ‘brain vascular resistance’/exp OR hemodynamic:ta,ab OR haemodynamic:ta,ab OR ((hemodynamic:ta,ab OR haemodynamic:ta,ab) AND (response:ta,ab OR responses:ta,ab)) AND ([article]/lim OR [article in press]/lim OR [conference abstract]/lim OR [conference paper]/lim OR [letter]/lim OR [note]/lim)
**Cochrane**	“lactates"[MeSH] OR “Monocarboxylic Acid Transporters"[MeSH] OR “L-lactate dehydrogenase"[MeSH] OR “lactic acid” OR lactate OR (lactic and (“acid receptors” OR “acid receptor”)) OR GPR81 OR HCAR1 OR (hydroxycarboxlic and “acid receptOR 1”) of (monocarboxylic and transport*):ti,ab,kw OR MCT OR MCTs OR MCT1 OR MCT2 OR MCT3 OR MCT4 OR “lactate dehydrogenase” OR LDH OR (hydroxypropanoic AND acid):ti,ab,kw AND "neurovascular coupling"[MeSH] OR “brain [MeSH] OR "neurons"[MeSH] OR "neuroglia"[MeSH] OR “astrocytes”[MeSH] OR "pericytes"[MeSH] OR neurovascular OR neurovasculature OR brain OR brains OR cerebral OR neuron OR neurons OR neural OR (nerve AND (cell OR cells)) OR astrocyte OR astrocytes:ti,ab,kw OR astroglia or astroglias or astroglial or microglia or microglial or pericyte OR pericytes OR (Rouget AND (cell OR cells)):ti,ab,kw AND "microvessels"[MeSH] OR endothelium, vascular"[MeSH] OR “hemodynamics”[MeSH] OR microvessel OR microvessels OR microvasculature OR microvascular OR ((Anastomoses OR anastomosis OR Anastomose) AND (Arteriovenous)) OR capillaries OR capillary OR Sinusoidal OR sinusoids OR sinusoid OR arteriole OR arterioles:ti,ab,kw OR venule OR venules OR endothelium OR endotheliums OR endothelial OR hemodynamic: OR haemodynamic OR ((hemodynamic OR haemodynamic) AND (response OR responses)): ti,ab,kw

Supplementary Table 1. Search strategies of the PubMed, Embase, and Cochrane databases.

Authors	Species (strain)	Age	n=	Intervention	Assessment Method	Finding
Spatz *et al.* 1978	Rat (Osbourne Mendel)	2-21 days<br>9 weeks	5-6<br>1	Uptake assay	Cerebral microvessel isolation<br><br>L-^{14}C-lactate (146.9 mCi/mol, 0.5-4mmol) in medium	Uptake far higher in post-natal brain L-[^{14}C] lactic acid was 12.4-fold higher in newborn vs adults.
Rieke and Cannon 1985	Rat (COB CD outbred)	3 months<br>10-12 months<br>25-27 months<br>31-33months	9<br>4<br>6<br>4	n/a	Histochemical analyses of LDH activity in cortical and basal ganglia vessels in freshly frozen slices. External diameters measured by ocular micrometer.	LDH present in all cortical vessels (moderate-strong reactivity) and very strong reactivity in largest vessel of 3month old animals.<br>Within basal ganglion and caudate putamen, LDH reactivity increased with vessel diameter.
Pirchl *et al.* 2006	Rat	10 days	n/a	Incubation with DL-lactate (10 nM-100 μM) for 7 days<br><br>Incubation with DL-lactate (10 nM-100 μM) for 4-6 days	Brain capillary endothelial cell culture. DAPI for cell survival<br><br>Organotypic slice of NBM Immunohistochemistry of cholinergic neuron.<br><br>Light microscope assessment	Stated that there was no effect of DL-lactate on slice or cell survival.<br>(data not shown)

Supplementary Table 2. Characteristics table of *in vitro* and *ex vivo* studies.
DAPI (4′,6-diamidino-2-phenylindole), LDH (lactate dehydrogenase), NBM (nuclear basalis of Meynert)

Authors	Species	Age	n=	Intervention	Assessment Method	Finding
Gordon *et al.* 2008	Rat (Sprague Dawley)	6-21 days	*See intervention*	1 mM lactate (PGE_2 n=5) (Arteriole diameter n=15) 100 µM indomethacin (n= 11) 2.5 mM oxamate (n=5) 200 µM iodoacetate (n=6)	Pharmacological intervention on hippocampal-neocortical slices<br><br>Lactate/PGE_2 assay<br><br>Two-photon microscopy and IR-DIC optics to measure arteriole diameter	Lactate increased extracellular levels of PGE_2 (+10.3 ± 0.4 pg/ml,25%) and increased arteriole diameter by 107.5 ± 3.8%).<br>Inhibition of dilation by COX, LDH, glycolysis inhibitors dilations mediated by astrocytic lactate.
Lauritzen *et al.* 2014	Mouse C57BL/6	6-8 weeks	*See method*	Antibodies against HCA1, MAP2, GS, and DAPI	Immunofluorescence histochemistry (n/a)	HCA1 present on: neurons, astrocytes, and capillaries in the cerebellum and the hippocampus.
	Mouse C57BL/6	4 months		HCA1 localization study	Electron microscopy Immunogold cytochemistry (n=3)	HCA1 identified on neurons, astrocytes and capillaries Concentrated at pre- and post- excitatory synapse over peri-synaptic densities. Present in endothelial cell membranes, twice as much than astrocytic endfeet

Supplementary Table 2 continued. COX (cyclooxygenase), GS (glutathione synthase), HCA1 (hydroxycarboxylic acid receptor 1) MAP2 (Microtubule associated protein 2), PGE_2 (Prostaglandin E_2),

Authors	Species	Age	n=	Assessment Method	Intervention	Finding
Miranda-Gonçalves *et al.* 2017	Human brain microvascular endothelial cells (immortalized, non-tumor)	min. 3 replicates		Glucose uptake assay: 2-NBDG<br><br>Mitochondrial activity: polarization via FACS<br><br>Western blotting: MCT1, MCT4, GLUT1, CD147, NF-κB, HIF-1α, HCA1, AMPK, pAMPK, AKT, pAKT, pERK, ERK<br><br>Immunofluorescence: MCT1, MCT4, GLUT1<br><br>BrdU Proliferation assay<br><br>Wound healing assay<br><br>Inhibition of MCT1 by AR-C155858<br><br>siRNA knockdowns of MCT1 and MCT4<br><br>Enzymatic colorimetric assay of lactate concentrations in media	Grown in high (25 mmol/l) and low (5.55 mmol/l) glucose media with addition of 20 mmol/l lactate<br><br>siRNA knockdowns of MCT1 and MCT4	High glucose: reduced glucose uptake, increased lactate uptake<br><br>Low glucose: increased lactate uptake, and increased mitochondrial activity, increased MCT1 expression, decreased GLUT1 expression, ~200% increase in proliferation, increase migration and capillary-like structures, increased NF-κB, HIF-1α, HCA1, AMPK/pAMPK.<br><br>Knocking down MCT1-4 reduced glucose consumption and lactate release (except MCT4), reduced proliferation, migration, and capillary density.

Supplementary Table 2 continued. (p)AKT ((phosphorylated)- RAC-alpha serine/threonine-protein kinase), AMPK (AMP-activated protein kinase), BrdU (bromodeoxyuridine), CD147 (Cluster of differentiation 147), (p)ERK ((phosphorylated)-Extracellular-signal regulated kinases), FACS (Fluorescence activated cell sorting), GLUT1 (Glucose transporter 1), HIF-1α (Hypoxia inducible factor 1-alpha), 2-NBDG ((2-(N-(7-Nitrobenz-2-oxa-1,3-diazol-4-yl)amino)-2-deoxyglucose), NFκB (Nuclear factor kappa,-light-chain-enhancer of activated B cells), siRNA (small interfering RNA)

.

**Authors**	**Species (strain)**	**Age Weight**	**n= (Sex)**	**Intervention**	**Assessment method**	**Finding**
Harper & Bell 1963	Dog (Mongrel)	n/a (n/a)	n/a (n/a)	2% lactic acid (0.22 mmol/l) administered via carotid artery	^{85}Kr injection/washout technique	No effect on CBF
Iwabuchi *et al.* 1973	Dog (Mongrel)	n/a 11-19 kg	11 (n/a	Reduction of MAP by hypovoleaemia110-30mmHg, (assumed autoregulation 110-50mmHg)	Lactate: levels measured in CSF, arterial blood and venous blood<br><br>CBF: dorsal sagittal sinus electromagnetic flowmetry	CSF lactate levels increased as pressure was lowered through autoregulation range (1.54 mmol/l to 1.78 mmol/l)
Bucciarelli & Eitzman 1979	Goat (Kid)	130-148 days gestation n/a<br><br>1-34 days postnatal n/a	21 (n/a)<br><br>20 (n/a)	1mmol/kg/min IV infusion of lactic acid over 5-10 min (5-10 mmol/kg)	Lactate: not stated<br><br>CBF: carbonized microsphere 15 ± 5μm labelled with ^{85}Sr, ^{51}Cr ^{141}Ce, ^{169}Yb.	Increase in CBF by 46 ± 2.1%<br>Arterial pH 7.22 ± 0.05
Hermansen *et al.* 1984	Dog (Mongrel)	6 ± 1 days 591 ± 50g	13 (7M 6F)	Induction of lactate acidosis. 2.2 mmol/kg over 5min and followed by continuous i.v. infusion to maintain pH at 7.20<br><br>Induction of hypotension reducing MABP by 30%	Lactate: not stated<br><br>CBF: Microsphere, baseline and 1hr after lactate addition	No change in CBF after 1hr infusion and CBF was preserved in hypovolemic measurements.

Supplementary Table 3. Characteristics of selected *in vivo* animal studies.
CSF (cerebral spinal fluid), MABP (Mean arterial blood pressure).

Authors	Species (strain)	Age Weight	n= (Sex)	Intervention	Assessment method	Finding
Powell *et al.* 1985	Dog (Mongrel)	Control<br>4 ± 2 days<br>550 ± 82g<br><br>Lactic acid<br>5 ± 3days<br>600 ± 235g	5 (n/a)<br><br>6 (n/a)	Intravenous infusion lactic acid 0.25 mmol/kg/min or 0.9% saline control for 15 min. (3.75 mmol/kg)	Lactate: fluorometric analysis of plasma<br><br>rCBF: 15 minutes post lactate infusion [^{14}C]-iodoantipyrine for 60sec arterial samples collected every 5sec for scintillation. 50µm sections for autoradiography	No effect on CBF once corrected for arterial $_{p}CO_2$ (48.3 ± 4.3mmHg)<br><br>Post-infusion blood lactate 9.2 ± 3 mmol/l lactate in<br>blood vs 2.5 ± 1.8 mmol/l in control.
Ong *et al.* 1986	Sheep	1-3 days	11 (n/a)	10 mg/kg lactic acid over 30 min (3.30 mmol/kg)<br><br>Phenylephrine (0.02%) induced increase in MABP	Lactate: none stated<br><br>CBF: ^{133}Xe (0.5mCi) washout technique	No direct effect on CBF but increased CBF with phenylephrine indicating potential effect on autoregulation
Hallström *et al.* 1990	Rat (Sprague Dawley)	n/a<br>290-310g	6 (n/a)	Electrical stimulation of the Nucleus Basalia of Meynert<br><br>Noxious mechanical stimulation of the hindpaw	Lactate: Microdialysis probe within parietal cortex<br><br>CBF: laser doppler probe on parietal cortex surface	Electrical and mechanical stimulation produced no change of lactate levels (333 ± 56 µM) with concurrent increase in CBF by 134 ± 4%.
McClung *et al.* 1990	Rabbit (New Zealand white)	n/a<br>2kg	15 (n/a)	Lactic acid infusion at 0.25,<br>0.5, 0.75 mmol/kg/h for 4 hours. (1 mmol/kg,<br>2 mmol/kg, 3 mmol/kg)	BBB permeability assessment via Polyethylene Glycol (PEG 400) IV infusion and measured in CSF<br><br>Pore Size calculated by using different MW size of PEG oligomers<br><br>PEG quantification by gas chromatography	Lactic acid increased the permeability of BBB<br><br><u>Control</u>:<br>Mean PEG M_w = 257 ± 8<br>Pore size (Å) = 7.3 ± 0.2<br>PEG mmol/l = 1.7 ± 0.5<br><br><u>Lactate</u><br>Mean PEG M_w = 294 ± 7<br>Pore size (Å) = 8.4 ± 0.2<br>PEG mmol/ = 4.0 ± 0.5

Supplementary Table 3 continued. BBB (Blood-brain-barrier), rCBF (relative CBF)

**Authors**	**Species (strain)**	**Age Weight**	**n= (Sex)**	**Intervention**	**Assessment method**	**Finding**
Young *et al.* 1991	Dog (mongrel) old	4-12 days 659 ± 68g	4 (physiology and NMR) (n/a)<br><br>Quantitative CBF Control n=8 Lactate n=3 (n/a)	L-lactic acid (33.3 mol/l) infused intravenously over 30 min followed by 30 min observation.	Lactate: ^{1}H NMR Spectra Arterial & venous blood<br><br>CBF: quantitative [^{14}C] iodoantipyrine (50 µCi)	Mean influx lactate into brain 0.43 µg/g/min, mean blood plasma lactate 30 mmol/l<br><br>Mean increase in CBF of 36 ± 9 % following lactate infusion, greatest in hindbrain and subcortical structures
Yanai *et al.* 1997	Rabbit (Albino)	n/a 2.6-3.2 kg	11 (M)	Hypoxia with FiO_2 3-11% 30 min baseline (30% FiO_2), 60 min hypoxia, 30 min recovery	CBF: Laser doppler flowmetry in right thalamus	Prolonged increase in rCBF during recovery period which, correlated with Lac/NAA ratio r=0.93, F=27.5
Zink *et al.* 1999	Pig (Yorkshire)	n/a 18-23 kg	Sham = 9 Intervention: TBI and ethanol= 13 (n/a)	Ethanol administered IV (2 g/kg, 20% ,30min)<br><br>1hr baseline data prior to TBI injury gives control data relative to sham.	Lactate: analyzed from cerebral venous blood<br><br>CBF: dye labelled microspheres<br><br>ICP also recorded.	Increase in ICP (mmHg). 6 ± 3- vs 10 ± 4 (p=0.048)<br><br>No change in CPP 87 ± 16 vs 78 ± 16 mmHg (p=0.47)<br><br>CBF only measured post-injury, no data available<br><br>Ethanol induced increase in cerebral venous blood lactate 1.27 ± 0.26 vs 2.20 ± 0.95 mmol /l (p=0.016)

Supplementary Table 3 continued.

FiO_2 (Fraction inspired oxygen), ICP (Intra-cranial pressure), NMR (Nuclear magnetic resonance), CPP (Cerebral perfusion pressure), TBI (Traumatic brain injury)

Authors	Species (strain)	Age Weight	n= (Sex)	Intervention	Assessment method	Finding
Ido *et al.* 2001	Rat (Sprague Dawley)	n/a<br>n/a	Saline n=7<br>L-lactate n=6<br>n/a	1.0 mmol/kg bolus	Lactate: arterial blood plasma samples<br><br>CBF: ^{125}I-desmethylimipramine iv	Unilateral stimulus of vibrissae with lactate bolus augmented CBF response by 100%<br>No effect on unstimulated cortex<br>Blood lactate not reported for CBF measurements
Ido *et al.* 2004	Rat (Sprague Dawley)	n/a<br>n/a	6-9 per group (M)	Visual checkerboard stimulus<br>1min 7.5Hz<br>unilateral or bilateral<br><br>1 or 2 mmol/kg lactate<br>15-20 sec bolus 1m prior to stimulation<br>2.5 min constant infusion<br><br>Visual cortex extracted	Lactate: arterial blood plasma samples<br><br>CBF: ^{125}I-labeled desmethylimipramine microspheres	Unilateral stimulus with bolus lactate: augmented visual cortex CBF by 11%<br>No effect of lactate unstimulated cortex.<br>Interhemispheric difference in CBF of 85%.<br><br>Bilateral stimulation with lactate infusion: increased CBF in visual cortex by 4%<br><br>Arterial blood plasma lactate levels (mmol/l)<br>Unstimulated baseline 1.2 ± 0.4;<br>During stimulation<br>2.6 ± 0.0 (1 mmol/kg); 3.5 ± 0.4 (2 mmol/l)<br><br>Arterial plasma lactate correlated positively with CBF r=0.652, $p<0.0001$
Provent *et al.* 2007	Rat (Wistar)	n/a<br>380-450 g	*See findings* (M)	NH_4Cl infusion iv.<br>2.5 mmol/kg over 4min (induces lactate release in brain).	Lactate: ^{1}H NMRS<br><br>CBF: ASL MRI	CBF increase after NH_4 infusion mirrors, brain lactate increase with a 5min delay. However, no direct link made between lactate and CBF<br><br>Brain Lactate (signal):<br>3.10 ± 0.35 (ratio peak/baseline, n=9)<br>% change NMRS peak, 210 ± 109, n=10<br><br>CBF: 2.16 ± 0.16 (ratio peak/ baseline, n=5)

Supplementary Table 3 continued.
ASL (Arterial Spin Labelling), NMRS (Nuclear Magnetic Resonance Spectroscopy).

Authors	Species (strain)	Age Weight	n= (Sex)	Intervention	Assessment method	Finding
E *et al.* 2013	Mouse C57BL/6	4 months n/a	54 (M)	Exercise: 2/day, for 5 days, 7 weeks<br>Treadmill: Lactate threshold<br>Warm up 3 min 15 m/min,<br>Weeks 1-3:<br>42 min 18 m/min<br>Week 4: 42 min 20 m/min<br>Week 5: 42 min 22 m/min<br>Week 6 42 min 25 m/min<br><br>Control n=11<br>Exercise n=12<br><br>Lactate: 18 mmol/kg ip. 1/day, 14 days<br>Lactate n=16<br>Saline n=15	Lactate: enzymatic assay of blood plasma<br><br>qRT-PCR: PGC-1α, PGC-1β;<br>PRC, NRF-1, TFAM, PCK1, PDK4, VEGF-A, HIF-1α, TNFα.<br><br>Brain mtDNA rt-PCR ND2,16s rRNA and 18s rRNA<br><br>Western blot: cytoplasmic and nuclear HIF-1α.	No effects of training/lactate on PGC-1α, PGC-1β,<br>NRF-1, or TFAM RNA.<br><br>Exercise (average peak of 175% plasma lactate relative to control group)<br>25% increase in PRC<br>20% increase VEGF-A<br>20% increase in 16s rRNA/18s rRNA ratio<br>30% decrease in TNFα<br><br>Lactate injection (average peak of 500% plasma lactate relative to baseline)<br>15% increase VEGFA<br>30% increase in PRC<br>15% increase in 16s rRNA/18s rRNA ratio<br>No change in TNFα<br><br>No effect on HIF-1α (transcription regulator of VEGF)<br><br>Correlation between VEGF-A and PRC mRNA r=0.665

Supplementary Table 3 continued.

HIF-1α (Hypoxia inducible factor 1-alpha), mtDNA (mitochondrial DNA), ND2 (NADH hydrogenase 2), NRF-1 (Nuclear respiratory factor-1), PCK1 (Phosphoenolpyruvate carboxykinase 1), PDK4 (Pyruvate dehydrogenase lipoamide kinase isozyme 4), PGC-1α (Peroxisome proliferator-activated receptor gamma coactivator 1-alpha), PGC-1β (Peroxisome proliferator-activated receptor gamma coactivator 1-beta), TNFα (Tumor necrosis factor alpha), TFAM (mitochondrial transcription factor A), VEGF-A (Vascular endothelial growth factor-A)

Authors	Species (strain)	Age Weight	n= (Sex)	Intervention	Assessment method	Finding
von Pföstl *et al.* 2012	Monkey Rhesus macaque	7-11 kg 4.6-12.5 kg	8 (7M 1F)	0,04mmol/kg/min lactate Infusion range of 0,15 – 0,6 M at 0,8 ml/min – 2,5ml/min<br><br>Control, PBS (n=5)	CBF: FAIR ASL (7T), (n=5)<br><br>BOLD fMRI visual boxcar chequerboard stimulus 48sec on/off while lactate infused<br><br>Low flow microdialysis iv (n=8)<br>2 µl/min, automated analysis	Positive baseline shift in BOLD signal (0.6 ± 0.2%), amplitude from 16-39%, during lactate infusion compared to baseline<br><br>Plasma lactate (mM) mean peak of 2.5 ± 0.9 (2.0-4.0) from mean baseline of 1.1 ± 0.5 (0.3-2.1)<br><br>Correlations between lactate and BOLD in 4 animals, r= 0.6, 0.52, 0.69, and 0.85, p-values not given<br><br>No observed effect on CBF<br>(detection threshold of method is approximately 5%)
Dostalova *et al.* 2017	Rabbit (New Zealand white)	n/a 1.5-2.0 kg	30 (15M) (15F)	Control (0.9%)<br>Sodium lactate<br>1.875 mmol/kg for 15min<br>Administered IV.	Lactate: blood plasma<br><br>CBF: side-stream dark-field via craniotomy up to 40min post bolus, evaluated "microvascular flow index"	No change in microvascular flow index up to 40min following infusion.<br><br>No difference in plasma lactate at 40min post infusion 5.1 ± 27 mmol/l and 5.0 ± 2.7 mmol/l (control and lactate groups respectively).

Supplementary Table 3 continued.

BOLD (Blood-oxygen level dependent), FAIR ASL (Flow Sensitive Inversion Recovery Arterial spin labelling), PBS (phosphate buffered saline)

Authors	Species (strain)	Age Weight	n= (Sex)	Intervention	Assessment method	Finding
Morland *et al.* 2017	Mouse (C57BL/6N)<br>WT<br>HCA1 KO/KO	7-9 weeks<br>(n/a)	WT n=21 (M10 F11)<br>KO=20 (M8 F12)	Exercise: Treadmill running<br>90% VO_2 max high intensity interval<br>2/day, for 5 days, 7 weeks<br>Treadmill: Lactate threshold<br>Warm up 10min 5m/min<br>10 high intensity interval training<br>2min active rest<br><br>Lactate:<br>18 mmol/kg sodium lactate in 0.9% saline, (pH7.4)<br>5/week for 7 weeks.<br><br>Hippocampal slice of HCA1 KO and WT, applied with 10 mM lactate or Krebs buffer,	Lactate: blood plasma analysis<br>Lactate (n=5)<br>Saline (n=3)<br><br>Capillary density and diameter in sensorimotor and cerebellar cortex measured by fluorescence microscopy<br>Control: WT n=7, KO n=5<br>Exercise: WT n=7, KO n=4<br>Lactate: WT n=7, KO n=6<br><br>Western blots of brain VEGF,<br>Control: WT n=4, KO n=6<br>Exercise: WT n=5, KO n=6<br>Lactate: WT n=6, KO n=6<br><br>Western blot of hippocampal slices ERK and PI3K/Akt known to control VEGF-A pathway	Both exercise and subcutaneous administration of lactate produced plasma lactate levels up to 10 mmol/l<br><br>Capillary density increased in sensorimotor cortex<br>(Exercise 1.15-fold; lactate 1.18-fold)<br><br>Capillary density increased in hippocampus cortex<br>(Exercise 1.5-fold; lactate 1.5-fold)<br><br>No change in capillary density in cerebellar cortex or<br>KO mice<br><br>Increase in hippocampal VEGF in<br>(Exercise 1.65-fold; lactate 1.75-fold)<br><br>No change in VEGF in cerebellar cortex or KO mice<br><br>Lactate induced phosphorylation of ERK and PI3K/Akt<br>via HCA1

Supplementary Table 3 continued.

ERK (Extracellular-signal regulated kinases), HCA1 (Hydroxycarboxylic acid receptor 1),PI3K/Akt (Phosphatidylinositol-4,5-bisphosphate 3-kinase/ Protein Kinase B). VEGF (Vascular endothelial growth factor), VO_2 Max (maximal oxygen consumption).

Authors	Age (Range)	n= (Sex)	Intervention	Assessment method	Finding
Stewart *et al.* 1988	31.6 ± 4.8 years (n/a)	5 (control) (1M 4F)	IV infusion of Sodium lactate 500mmol/kg,<br>10 ml 20-30 min<br>Saline baseline control	CBF: Inhaled ^{133}Xe CAT	Mean increase in CBF 20.4% and 19.7% in left and right hemispheres respectively.<br>CBF in lactate group in ml/100g<br>(% change rel. to control)<br>Left Frontal, 93 ± 8 (19.7%)<br>Right Frontal, 86 ± 8 (15.5%)<br>Left Occipital 87 ± 13 (9.0%)<br>Right Occipital 92 ± 8 (6.8%)
Reiman *et al.* 1989	25 years (22-30)	15 (7M 8F)	IV infusion 500 mmol/l at 0.5 mg/kg/min over 20-30 min<br>(89.2 – 113 mmol/kg)	CBF: PET 50-70 mCi [^{15}O] water	No effects of lactate on CBF in control subjects
Mintun *et al.* 2004	24±4 years (20-27)	7 (3M 4F)	IV lactate bolus 1 mmol/kg in 20 ml over 30 sec administered following intra-subject saline control<br><br>CBF responses to lactate measured in visual cortex at rest and stimulation	CBF: PET 50mCi [^{15}O] water<br><br>Lactate measured in arterial blood<br>plasma	Lactate did not modulate resting CBF globally, nor in the visual cortex<br><br>Lactate augmented the CBF response by 38-53%.<br><br>CBF correlated with lactate:pyruvate ratio r=0.947, p=0.01)<br>and lactate (r=0.748, p=0.053); but not pyruvate (r=0.216, p=0.643).<br><br>Plasma lactate baseline 0.8±0.03 mmol/l (rest) and 1.6±0.08 mmol/l (stimulus) increased to10.7 ± 2.8 mmol/l (rest) and 9.8±2.4 mmol/l (stimulus) after lactate<br>Infusion
Lin *et al.* 2010	(n/a) 22-38 years	12 (7M 5F)	Chequerboard visual stimulus at 4Hz, 8Hz, and 16Hz. 4min on/off	Simultaneous VASO (CBV), ASL (CBF) and BOLD at 3T. Cerebral lactate measured by ^{1}H NMR spectra in V1	%ΔCBF correlated with %ΔLactate (r=0.91)<br>but not as well with calculated $CMRO_2$ (r=0.64).

Supplementary Table 4. Characteristics of selected human studies. ASL (Arterial spin labelling), $CMRO_2$ (Cerebral metabolic rate of oxygen), CAT (Computed axial tomography), NMR (Nuclear Magnetic Resonance), PET (Positron Emission Tomography), VASO (Vascular space occupancy). V1 (Primary visual cortex).
